# Early enteral feeding in the septic critically ill patient: evaluation of our feeding protocol

**DOI:** 10.1186/cc13616

**Published:** 2014-03-17

**Authors:** M Theodorakopoulou, I Dimopoulou, S Karambi, A Strilakou, A Diamantakis, S Orfanos, A Armaganidis

**Affiliations:** 1 University Hospital of Athens Greece 'Attikon', Attiki, Greece

## Introduction

It has been established that early enteral nutrition in critically ill patients improves overall outcome and mortality. In our unit, feeding protocols were established based on the ESPEN recommendations and have been implemented for the last 2 years. The purpose of this study was to evaluate the compliance of our septic patients' nutritional approach with our feeding protocols.

## Methods

A prospective study was done on a 24-bed mixed ICU over a period of 18 months. Eighty-three patients ≥18 years were included in the study. All patients were dependent on mechanical ventilation and met the CCM criteria for sepsis upon admission to the ICU. APACHE II score, SOFA score, weight, BMI and nutritional status were calculated. Patients were initiated for enteral feeding based on the established feeding protocol within 48 hours of admission. The feeding status of the patient was recorded on the start day (D0), day 3 (D3) and day 7 (D7). Factors affecting the feeding process and its progression were also recorded

## Results

The patient mean age was 71.4 ± 12.2. LOS in the ICU was 9 to 21 days. Based on BMI, 18% of the patients were malnourished upon admission. APACHE II was 26 ± 7.8 and SOFA was 9.2 ± 4.6. The mortality rate was 42.5%. Enteral nutrition started early in 64 (77.1%) of the patients (D0), on day 3 (D3) 29 (45.31%) patients met their caloric goals and on day 7 (D7) only 18 (28.1%) patients achieved their caloric goals. Discontinuation of enteral feeding was mainly due to procedures, whereas late start and/or decreased hourly intake were due to GI complications, GI intolerance, excessive diarrhoea and hemodynamic instability. There was no association between compliance with the feeding protocol and the LOS, nutritional status, severity or disease progression.

## Conclusion

Although the initiation of early enteral feeding seems adequate for a good number of septic patients on D0, is still far off for a significant percentage of those patients on D3 and is even worse on D7. The caloric goal achievements were better on D3 but very suboptimal on D7. There was no association, however, between nutritional status and compliance with the feeding protocols. It is therefore mandatory to follow daily the nutritional therapy of the septic patient and not rely only on the feeding protocols.

